# Preoperative Behavioural Intervention versus standard care to Reduce Drinking before elective orthopaedic Surgery (PRE-OP BIRDS): protocol for a multicentre pilot randomised controlled trial

**DOI:** 10.1186/s40814-018-0330-4

**Published:** 2018-08-16

**Authors:** Christopher Snowden, Ellen Lynch, Leah Avery, Craig Gerrand, Eilish Gilvarry, Nicola Goudie, Catherine Haighton, Lesley Hall, Nicola Howe, Denise Howel, Elaine McColl, James Prentis, Elaine Stamp, Eileen Kaner

**Affiliations:** 1 The Newcastle upon Tyne Hospitals NHS Foundation Trust, Freeman Hospital, Freeman Road, Newcastle upon Tyne, NE7 7DN UK; 2 Institute of Health & Society, Newcastle University, Baddiley-Clark, Richardson Road, Newcastle upon Tyne, NE2 4AX UK; 3 Institute of Cellular Medicine, 4th Floor, William Leech Building, Medical School, Newcastle University, Newcastle upon Tyne, NE2 4HH UK; 4 Newcastle Addictions Service, Northumberland Tyne and Wear NHS Foundation Trust, Plummer Court, Carliol Square, Newcastle upon Tyne, NE1 6UR UK; 5 Newcastle Clinical Trials Unit, Newcastle University, 1-4 Claremont Terrace, Newcastle upon Tyne, NE2 4AE UK; 6 Department of Social Work, Education & Community Wellbeing, Northumbria University, Room B125, Coach Lane Campus West, Newcastle upon Tyne, NE7 7XA UK

**Keywords:** Alcohol, Perioperative, Brief behavioural intervention, Behaviour change, Optimisation, Surgery, Pilot randomised controlled trial

## Abstract

**Background:**

Evidence suggests that increased preoperative alcohol consumption increases the risk of postoperative complications; therefore, a reduction or cessation in alcohol intake before surgery may reduce perioperative risk. Preoperative assessment presents an opportunity to intervene to optimise patients for surgery. This multicentre, two-arm, parallel group, individually randomised controlled trial will investigate whether a definitive trial of a brief behavioural intervention aimed at reducing preoperative alcohol consumption is feasible and acceptable to healthcare professionals responsible for its delivery and the preoperative elective orthopaedic patient population.

**Methods:**

Screening will be conducted by trained healthcare professionals at three hospitals in the North East of England. Eligible patients (those aged 18 or over, listed for elective hip or knee arthroplasty surgery and scoring 5 or more or reporting consumption of six or more units on a single occasion at least weekly on the alcohol screening tool) who enrol in the trial will be randomised on a one-to-one non-blinded basis to either treatment as usual or brief behavioural intervention delivered in the pre-assessment clinic. Patients will be followed up 1–2 days pre-surgery, 1–5 days post-surgery (as an in-patient), 6 weeks post-surgery, and 6 months post intervention. Feasibility will be assessed through rates of screening, eligibility, recruitment, and retention to 6-month follow-up. An embedded qualitative study will explore the acceptability of study methods to patients and staff.

**Discussion:**

This pilot randomised controlled trial will establish the feasibility and acceptability of trial procedures reducing uncertainties ahead of a definitive randomised controlled trial to establish the effectiveness of brief behavioural intervention to reduce alcohol consumption in the preoperative period and the potential impact on perioperative complications.

**Trial registration:**

Reference number ISRCTN36257982

## Background

Preoperative alcohol consumption increases the risk of postoperative complications following major surgery [1, 2] and specifically orthopaedic surgery [3, 4]. Postoperative complications are associated with increased length of hospital stay, delayed patient recovery, reduced well-being [5] and a two- to threefold increase in hospital costs [6]. Even minor postoperative complications reduce functional independence [7] and cause significant decrease in longevity [5, 8].

In the elective orthopaedic surgical population, average rates of mortality and morbidity following primary total hip and knee replacement are 0.2–0.6% [9–11] and 4–6% respectively [9, 12]. In terms of major surgery, these rates are relatively low. However, primary arthroplasty already represents a high volume type of major surgery [13]. As life expectancy continues to increase worldwide [14], both the shift in ageing demographics and the natural history of arthritic disease will inevitably lead to a greater number of older patients undergoing major elective orthopaedic surgery. With advancing age also comes an increase in the number of significant comorbidities [15]. This combination of high-volume surgery in an ever-increasing population of older patients with multiple comorbidities is a potent risk for an increase in postoperative complications [16, 17] and will become a major public health burden [18] if specific interventions to reduce perioperative risk are not developed.

‘Prehabilitation’ refers to a group of interventions, integrated into the clinical pathway before a proposed surgical procedure, aimed at both reducing imminent patient risk and promoting lasting beneficial effects on perioperative recovery and outcome. The benefits of preoperative anaemia correction, collaborative decision making [19], multimodal pulmonary training regimes, improving nutrition while encouraging physical activity, and promoting structured exercise regimes are improving patient outcomes [20–26]. Lifestyle modification is a key component of the value proposition for perioperative care [27]. Alongside smoking cessation [28] alcohol reduction prior to surgery is an important prehabilitative goal.

Among patients undergoing total hip or knee arthroplasty, those identified as having alcohol misuse showed significantly higher incidences of 12 out of 15 complications assessed [29] with an overall complication rate of 33.5% among alcohol misusers compared to 22.6% in their non-alcohol misusing counterparts. This population were also found to have significantly longer stays in hospital [29]. Further to this, a study of men undergoing total joint arthroplasty reports a 29% increase in the number of complications for every additional point on the Alcohol Use Disorders Identification Tool (AUDIT) screening tool [3]. Therefore, cessation or even reduction in alcohol intake before surgery may reduce perioperative risk [3, 30] and could have public health benefit if behaviour change continues post operatively. Alcohol screening using validated screening tools has been shown to have good patient acceptance [3, 30–33]. In addition, where a dedicated screening tool (AUDIT) [34]) has been used in the European preoperative setting [35], there was an increased detection rate of preoperative alcohol use when compared with clinician assessment. However, in general, these methods have not been introduced into UK elective surgical pathways.

Good quality evidence exists for the effective delivery of brief behavioural interventions for the reduction of alcohol intake in primary care [36–39], with those who received brief intervention reporting lower weekly alcohol consumption compared to control participants (mean difference − 20 g, 95% confidence intervals (CI) − 28 to − 12) [38]. Evidence for the effectiveness of such interventions in emergency departments [40, 41] and hospital wards [42] is also rapidly growing. In perioperative care, Rosenberg et al. [41] reported reduced drinking and fewer postoperative complications among alcohol dependent patients who withdrew from alcohol consumption and remained abstinent supported by disulfiram therapy in the month before surgery compared to those who received treatment as usual. Further to this, a meta-analysis [1] of preoperative interventions including pharmacological components found a significant reduction in complication rates (odds ratio (OR) 0.22, (CI) 0.08 to 0.61, *P* = 0.04). However, less evidence exists regarding the effectiveness of behavioural interventions to reduce alcohol consumption before surgery [1] and findings from such studies are mixed. Specifically, in unpublished data, cited in a Cochrane review [1], Tonnesen et al. reported that intensive motivational counselling, delivered as weekly brief interviews and supported by disulfiram therapy, aimed at 3 months of preoperative alcohol cessation, reduced alcohol consumption in all patients in the intervention group. In contrast, Shourie et al. [43] showed no effect on drinking outcomes of a brief behavioural intervention. The discrepancies in findings between these two trials may be due to methodological differences, including the utilisation of both pharmacological and behavioural interventions and length and intensity of the interventions. Tonneson and colleagues [1] employed a more in-depth intervention delivered over a 3-month period before surgery while Shourie and colleagues [43] employed a brief intervention with preoperative review restricted to < 7 days before operation, a factor which the authors subsequently quoted as the major reason for intervention failure. With such variation in findings, more evidence regarding the effectiveness of behavioural interventions to reduce alcohol consumption in the preoperative period is required. Future trials should aim to deliver intentions with greater than 7 days for the implementation of behaviour change, something which this pilot randomised controlled trial (RCT) will aim to achieve by delivering interventions at the preoperative assessment appointment.

With previous trials having been conducted outside of the UK and findings from these trials being inconclusive, it is considered important to optimise intervention and research methods and assess the feasibility of conducting a full RCT which would then assess the effectiveness of behavioural interventions to reduce alcohol consumption in the preoperative period. Initial feasibility work was conducted at one centre between April 2016 and February 2017 with the aim of optimising the training, screening and intervention techniques prior to the larger multicentre pilot RCT. As such, the feasibility study did not include randomisation or follow-up assessments. A group training session covering the delivery of alcohol screening and brief behavioural intervention was provided to healthcare professionals (HCPs) employed in preoperative assessment. Screening (using the AUDIT C) and recruitment of eligible patients was then conducted with recruited patients completing the full AUDIT and receiving the behavioural intervention. Finally, qualitative interviews were conducted with HCPs and patients to explore the acceptability of the proposed brief intervention and training methods, patient participants were also presented with copies of the proposed pilot RCT follow-up questionnaires and asked to provide their opinion on these. A total of nine HCPs consented to be involved in the study with four going on to deliver interventions. Subsequent screening data reveal approximately 29% of patients listed for elective hip or knee arthroplasty score ≥ 5 on the AUDIT C and almost 16% report consumption of six or more standard drinks on a single occasion at least weekly. Fifteen patients were recruited to the study. Intervention sessions were recorded and assessed for fidelity of delivery with feedback provided to the HCPs. This work identified and addressed some implementation issues, detailed in the “Discussion” section. Follow-up interviews (HCPs *n* = 3; patients *n* = 11) found that proposed screening and intervention techniques (i.e. behaviour change and brief motivational techniques) were acceptable to both patients and healthcare professionals delivering the intervention. Further to this, screening and intervention could be conducted without impacting negatively on patient care. Focus groups (*N* = 3) conducted with HCPs (*N* = 19) from across the three centres established the characteristics of treatment as usual in preoperative assessment. Fidelity assessments and HCP interviews were utilised to optimise the training ahead of the pilot RCT. This pilot randomised controlled trial (RCT) aims to assess the feasibility and acceptability of HCP-delivered structured alcohol screening followed by brief behavioural intervention in the preoperative assessment clinic for patients undergoing primary elective hip or knee arthroplasty. Specifically, rates of recruit to a randomised trial of the brief behavioural intervention, retention rates at 6-month follow-up and completion rates for outcome measures will be assessed.

## Methods/design

### Design

A multicentre, two-arm (treatment as usual (TAU) vs brief behavioural intervention), parallel group, individually randomised controlled trial is used. A summary of the trial is displayed in Table 1.

**Table 1:** Trial summary

Data category	Information
Primary registry and trial identifying number	ISRCTN36257982
Date of registration in primary registry	06/01/2016
Secondary identifying numbers	REC: 17/NE/0093 Funder Reference: 14/42/01
Source(s) of monetary or material support	National Institute for Health Research Health Technology Assessment
Primary sponsor	Newcastle upon Tyne Hospitals NHS Foundation Trust
Secondary sponsor(s)	N/A
Contact for public queries	Miss Nicola Goudie Newcastle Clinical Trials Unit Newcastle University 1–4 Claremont Terrace Newcastle upon Tyne NE2 4AE United Kingdom
Contact for scientific queries	Miss Nicola Goudie Newcastle Clinical Trials Unit Newcastle University 1–4 Claremont Terrace Newcastle upon Tyne NE2 4AE United Kingdom
Public title	Preoperative Behavioural Intervention to Reduce Drinking before elective orthopaedic Surgery
Scientific title	Preoperative Behavioural Intervention to Reduce Drinking before elective orthopaedic Surgery
Countries of recruitment	England
Health condition(s) or problem(s) studied	Preoperative alcohol consumption
Intervention(s)	Intervention: Preoperative Brief Behavioural Intervention to reduce or cease drinking in elective orthopaedic patients Comparator: Treatment as Usual
Key inclusion and exclusion criteria	Ages eligible for study: ≥18 years Sexes eligible for study: both Accepts healthy volunteers: no Inclusion criteria: adult patient (≥ 18 years), listed for primary elective joint (hip or knee) arthroplasty, AUDIT C score ≥ 5 or report consuming 6 units or more in one session at least weekly with the capacity to provide informed written consent and able to write and converse in English Exclusion criteria: Patients likely to undergo sequential joint replacements within the duration of the study, those displaying current (active) withdrawal from alcohol, those with severe psychiatric disorder requiring medical treatment, cognitive impairments or dementia impacting ability to interact with the intervention
Study type	Interventional Allocation: randomised (1:1) Intervention model: parallel assignment Masking: non blinded
Date of first enrolment	N/A
Target sample size	80
Recruitment status	Recruitment to commence June 2017
Primary outcome(s)	Number of patients screened, and the percentages of eligible patients recruited and retained at 6-month follow-up.
Key secondary outcomes	Alcohol consumption: full AUDIT score Health Related Quality of Life: The EQ-5D Major and minor postoperative complications: Clavien-Dindo classification; Postoperative Morbidity Survey (POMS) Joint functionality: Western Ontario and McMaster Universities osteoarthritis index (WOMAC) Functional Assessment score.

### Aim

The primary aim of this pilot RCT is:

To estimate rates of patient eligibility, recruitment and retention at 6 months post-assessment in order to assess the feasibility of proceeding to a definitive RCT.

The secondary objectives are:

- To train healthcare professionals (HCPs) in the delivery of structured screening and brief behavioural intervention to eligible patients in the preoperative assessment setting.
- To assess completion rates for all data collection tools including measures of alcohol consumption, quality of life and joint functionality.
- To establish response variability of proposed outcome measures for a definitive trial, which will include drinking status and quality of life
- To estimate rates of secondary outcomes and perioperative complication rates including bleeding and infections
- To explore the acceptability of intervention and research methods with HCPs and patients through qualitative interviews.

While this pilot RCT is primarily concerned with recruitment and retention, a definitive RCT would have the primary objective of evaluating the effectiveness of brief behavioural intervention in reducing perioperative alcohol consumption. Secondary objectives would include assessing any resultant changes in secondary outcomes of alcohol perioperative complication rates and quality of life as well as longer-term changes in alcohol consumption at 6 months post intervention.

### Setting

The RCT will be conducted across three secondary care hospital clinics, in North East England, dedicated to preoperative assessment of patients before elective major surgery. Each has an orthopaedic preoperative surgical care pathway of 6 to 10 weeks from preoperative assessment to surgery. Across the three sites, approximately 30 HCPs are employed in the preoperative assessment clinics and a combined total of approximately 4000 hip or knee arthroplasties are conducted each year. Based on AUDIT C screening results from the feasibility study, this would provide a pool of approximately 1160 eligible patients.

### Participants—patients

The pilot trial will aim to recruit 80 (40 in each trial arm) patients. Patient participants will be adults aged 18 years and over, listed for elective primary hip or knee arthroplasty who screen positively for increased risk drinking (AUDIT C score ≥ 5 or report consuming 6 units or more in one session at least weekly) with the capacity to provide informed written consent and able to write and converse in English. Patients likely to undergo sequential joint replacements within the duration of the study, those displaying current (active) withdrawal from alcohol, those with severe psychiatric disorder requiring medical treatment, cognitive impairments or dementia impacting ability to interact with the intervention will be excluded in this RCT.

### Participants—healthcare professionals

Across the three sites, HCPs employed in the preoperative assessment clinics will receive training on study procedures, screening patients using the AUDIT C tool and completion of study baseline measures. In addition, a subset of HCPs (minimum two per centre) will be trained to deliver the brief behavioural intervention.

The intervention is detailed in Table 2. The content of the intervention materials have been defined in terms of specific behaviour change techniques (BCTs) that are the ‘active ingredients’ of the brief behavioural intervention. Group training sessions, delivered by the research associate (EL) and a health psychologist (LA), will be provided to HCPs at each hospital site. Training will cover delivery of the intervention including use of specific brief motivational techniques, and each HCP will also receive a training manual to support the formal training session. This training approach aims to equip HCPs with the knowledge and skills they need to conduct screening with a validated screening tool and deliver brief behavioural interventions to motivate and support patients to either reduce their drinking to low-risk levels or abstain from drinking in the perioperative period (the proposed objective for a definitive RCT).

**Table 2:** TIDieR description of intervention

Item	Details	Page
Name	Preoperative Behavioural Intervention to Reduce Drinking before elective orthopaedic Surgery (PRE-OP BIRDS)	1
Why	Preoperative alcohol consumption is related to increased risk of postoperative complications. The aim of the intervention is to support patients to reduce or cease alcohol consumption prior to elective orthopaedic surgery.	7–11
What	Materials: Healthcare professionals (HCPs) are trained to screen for increased risk alcohol consumption and to deliver the following intervention materials:	14
	• Brief Advice Sheet • Brief Intervention Sheet • Patient Information Leaflet	17–19
	Training also covers use of brief motivational techniques to increase motivation for change. Training of healthcare professionals is supported by a training manual. Procedures: The intervention is delivered over two sessions (the second is optional). The first session involves provision of 5 min of structured advice that aims to increase motivation using the ‘brief advice sheet’. This is followed by 15–25 min of behaviour change intervention using the brief intervention sheet. This intervention targets volitional aspects of behaviour change. The aim of the second optional booster session is to review and/or revise behavioural goals, provide feedback on performance and discuss self-monitoring to increase self-efficacy. This session is also designed to allow those individuals who have showed an initial intention to make changes, but who have not formally set behavioural goal(s) and plans to do so if desired.	17–19
Who provided	HCPs employed in the Preoperative assessment clinics who have received training in the delivery of screening and brief behavioural intervention.	14
How	The initial intervention session is delivered face to face during routine clinics. The second session, an optional booster session will be delivered either face to face in clinic or by telephone depending upon patient preference. All intervention sessions are delivered one to one.	17–19
Where	Intervention sessions will be delivered during routine preoperative assessment clinics. Where the patent opts to receive a booster session by telephone the HCP delivering the session will call from the preoperative assessment clinic.	13
When and how much	The first session involving delivery of brief advice and brief behavioural intervention will last approximately 30 min and will be delivered during routine pre-assessment clinics once all clinical procedures have been completed. The second optional, ‘booster’ session will last approximately 20 min and will be delivered around 1 week before surgery.	17–19
Tailoring	Intervention materials incorporate specific behaviour change techniques that target intention formation and enactment of behaviour change (e.g. information on health consequences, social support, goal setting behaviour, problem solving, restructuring the physical environment). HCPs are trained to use these techniques to tailor the intervention to the needs and preferences of the individual patient. For example, providing information relevant to and requested by the patient and supporting them to set meaningful and realistic goals that fit in with their own specific circumstances. Use of brief motivational techniques by HCPs allows them to determine level of motivation to change and tailor the intervention to target motivation or volition at the appropriate times.	17–19
How well	Consultations with participating patients will be audio recorded to allow an assessment of skill acquisition and fidelity of delivery of the intervention post-training. The aim is to improve fidelity of delivery via provision of feedback to HCPs including aspects of intervention delivery that went well and where they could improve. Feedback is provided following delivery of the intervention by each HCP to patients 2 and 4.	14–15 26–27

Each preoperative assessment HCP who has been trained will aim to conduct a minimum of four patient consultations where the intervention is delivered in order to facilitate full engagement with intervention delivery and allow feedback on fidelity of delivery. Audio recordings will be made of all intervention sessions where the patient provides consent. All recordings will be transcribed verbatim before being deleted from the recording device. All anonymised transcripts will be assessed for fidelity of delivery by two members of the research team (EL and LA) using a standardised checklist of 18 BCTs and 5 behaviour change counselling techniques. As the intervention is designed to allow tailored delivery, assessors will first identify if each item was appropriate before assessing if it was delivered. Feedback (constructed by the research associate (EL) and health psychologist (LA)) regarding skill acquisition and fidelity of delivery will be provided to HCPs for the second and fourth intervention sessions they deliver before any subsequent consultations where the intervention is delivered take place. Where patients in sessions 2 and/or 4 do not consent to recording, fidelity of delivery assessments will be conducted and feedback provided for the next recorded session (where a patient does provide consent). If required (i.e. where fidelity of delivery falls below the 80% threshold, based on the percentage of items assessed as ‘appropriate’ that were identified as delivered), the research associate (EL) will provide an additional, one-to-one, face-to-face training session, lasting up to 1 h to focus on components of the intervention that were omitted when it may have been appropriate to deliver them.

### Screening, consent and randomisation

**Fig. 1:**
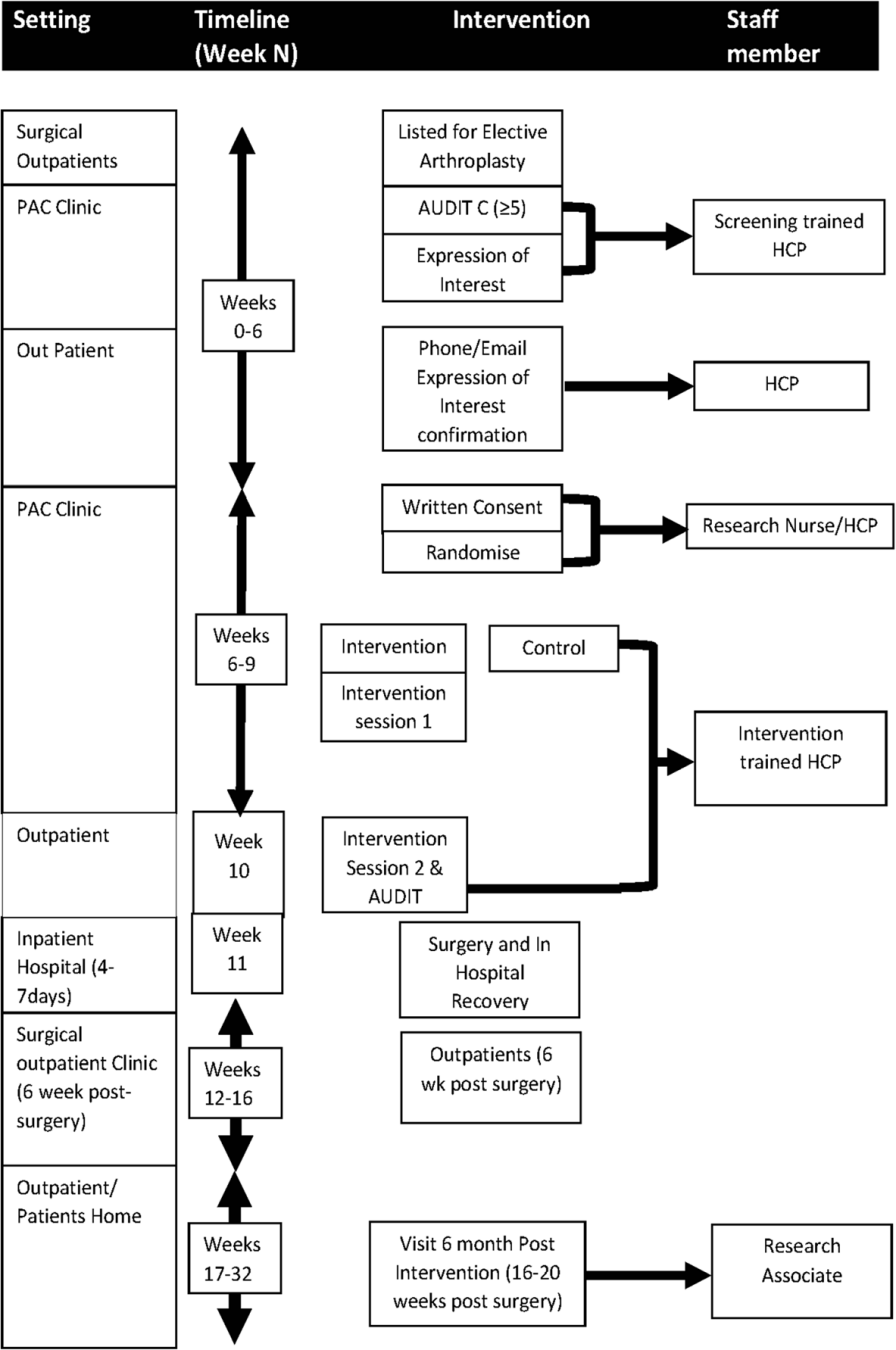
Trial process diagram

The study process is shown in Fig. 1. Initial screening for alcohol consumption using the AUDIT C will be conducted either in the outpatient clinic or by telephone following assessment and listing for elective orthopaedic surgery. Screening will be performed by an HCP trained in AUDIT C use and scoring. Screening will be conducted in an appropriate environment offering the required privacy. The allocated HCP will complete the AUDIT C with each patient listed for major joint arthroplasty. The AUDIT C score will be assessed immediately for eligibility to the study.

Eligibility for the study will be confirmed if the elective surgical patient screens positively for increasing risk drinking (AUDIT C score ≥ 5) or reports at least weekly drinking of 6 or more units in one session (as judged by question 3 on AUDIT C). The trial will be discussed with eligible patients and those that express an interest in participating will be provided with a copy of the participant information sheet. They will also be asked for permission for the study team to contact them to discuss the study further and their preferred method of contact. Expressions of interest, contact information and preferred method of contact will be recorded in writing by the HCP on the expression of interest form. The patient will then be allowed time to consider their involvement in the study (minimum 24 h). After this time, the site will contact the patient to confirm positive expression of ongoing interest. If the patient confirms they are still interested, the pre-assessment clinic will be informed of the patient’s likely participation to facilitate waiting list and preoperative assessment clinic coordination for the patient’s visit. Patients scoring AUDIT C < 5 will receive positive feedback on their low-risk drinking status.

At the preoperative assessment clinic, a member of the research team (not involved in screening or delivering the intervention) will initially see the patient. The researcher will check whether the patient is still interested in participating in the study, and if so, the patient will be asked to sign the informed consent form. Once consent has been obtained, the researcher will access the Newcastle Clinical Trials Unit (NCTU) secure web-based randomisation system where they will enter the patient’s initials and date of birth. The randomisation system will then allocate the patient to either the behavioural intervention or treatment as usual groups on a 1:1 basis. Allocations are built into the randomisation system as part of study setup, and users accessing the system to randomise have no control over the allocation. This trial is not blinded.

### Intervention

The brief behavioural intervention will be delivered over two sessions—the first session which will take place in the clinic will last approximately 30 min and will be delivered following pre-assessment in the pre-assessment clinic. The second session, an optional booster session, will be delivered over the phone or in the clinic dependent on patient preference and will last approximately 20 min. This will be delivered approximately 1 week before surgery.

Following preoperative assessment, the patient and HCP will complete the baseline assessments shown in Table 3 (full 10-item AUDIT [34], EQ-5D [44, 45], Western Ontario and McMaster Universities Osteoarthritis Index (WOMAC) [46]); the HCP will then deliver the first intervention session. This session will provide participants with 5 min of simple, structured advice regarding alcohol consumption including specific feedback on risk, normative comparison, the benefits of reducing alcohol intake, strategies that could be used to reduce drinking and a personal target for reduced drinking (set by the patient). This component of the intervention specifically targets motivation. This will be followed by 15–25 min of behaviour change intervention targeting alcohol reduction and cessation and specifically focuses on volition. Intervention materials incorporate specific behaviour change techniques that target intention formation and enactment of behaviour change (e.g. information on health consequences, social support, goal setting behaviour, problem solving, restructuring the physical environment). HCPs are trained to use these techniques to tailor the intervention to the needs and preferences of the individual patient, for example, providing information relevant to and requested by the patient and supporting them to set meaningful and realistic goals that fit in with their own specific circumstances. Use of brief motivational techniques by HCPs allows them to determine the level of motivation to change and tailor the intervention to target motivation or volition at the appropriate times. Three intervention tools will be utilised:

- The Brief Advice (BA) tool will act as a visual aid for communicating information and will provide participating HCPs with prompts on how to structure and deliver this advice to participating patients.
- The Brief Behaviour Change intervention (BI) tool will be used by HCPs to guide discussion, goal setting and problem solving with participating patients and to record aspects of the intervention (e.g. goals and plans) to prompt behaviour outside of the clinical setting.
- The Patient Information Leaflet, based on the based on the Department of Health’s ‘Drinking and you: How much is too much?’ booklet [47], will be introduced and given to patients at the end of the intervention session along with copies of the BA and BI.

**Table 3:** Schedule of enrolment, interventions and assessments

Time point	Study period						
	Screening	Visit 1 6–8 weeks pre-surgery	Visit 2 1–2 weeks pre-surgery (intervention arm only)	Visit 3 1 and 3 days pre-surgery	Visit 4 Up to 5 days post-surgery	Visit 5 6 weeks post-surgery	Visit 6 6 months post intervention
Enrolment:							
AUDIT C	X						
Expression of Interest	X						
Consent		X					
Randomisation		X					
Interventions:							
Intervention		X					
Booster			X				
TAU		X					
Assessments:							
AUDIT Baseline		X					
EQ-5D Baseline		X					
AUDIT Outcome			X	X		X	X
EQ-5D Outcome						X	X
WOMAC		X			X		
POMS					X		
Clavien-Dindo					X		
Interview							X

The BA and BI tools were based on the ‘How much is too much?’ Simple Structured Advice, and Extended Brief Intervention tools originally produced as part of the World Health Organization collaborative study on alcohol screening and brief intervention [48], before being adapted for use in the UK [49] and updated [37].

Patients who consent to be contacted for the second ‘booster’ session will be contacted approximately 1 week before surgery to arrange receipt. This will take place either over the telephone or in clinic at the patient’s preference. The HCP delivering the booster session will complete the full AUDIT questionnaire with patients and will aim to review or revise behavioural goals, provide feedback on performance and discuss self-monitoring to increase self-efficacy. The aim of the booster is to reinforce behaviour change by providing an opportunity for participants who had not set a goal or made a plan to reduce their drinking in the initial session the opportunity to do so. For those participants who had set a goal, the booster offers an opportunity to reflect on their experiences since the initial session, receive feedback on their progress and revise or amend their goal if necessary. Such sessions have previously been employed to address limitations presented by busy clinical settings where clinician time may be restricted and participants attention may be elsewhere (such as on their medical care) [50]; however, as booster sessions are not often well attended [51] and findings regarding the added benefit of booster sessions are mixed [52, 53], it was decided that these sessions would be optional rather than required.

### Comparator

Patients allocated to TAU will not receive the intervention but will complete study baseline measures. As part of the feasibility study, focus groups (*N* = 3) were conducted with HCPs (total *N* = 19) from across the three hospital sites to characterise TAU. As part of the pre-assessment, all patients are asked about their alcohol consumption. Questions cover whether the patient consumes alcohol and, if so, aim to establish the number of units consumed each week. Patients considered by the preoperative assessment clinic nurse to be ‘heavy drinkers’ or those identified through blood and/or liver function tests are routinely referred to the consultant anaesthetist for anaesthetic review to ensure patient safety during anaesthesia.

Additionally, there is specialist support available for patients to reduce or cease alcohol consumption at all three participating centres. However, findings from the focus groups identified that in the preoperative assessment setting there is currently no standard process for identifying and referring patients to these services with identification being left to the judgement of the individual HCP. Further to this, referrals to these services from preoperative assessment HCPs were very low as a result of a lack of knowledge regarding how to refer patients to these services and, at one site, a requirement to ask patients if they wished to receive further support an offer which staff reported patients consistently declined. While leaflets about alcohol consumption were visibly available in the waiting areas of the preoperative assessment clinics, HCPs did not report routinely providing these to patients. In addition, these are hospital-wide services, not specific to the pre-assessment department and information or advice is not delivered by preoperative assessment clinic staff.

### Measures

The measures completed at each time point are displayed in Table 3.

The primary feasibility outcomes of this pilot trial will be the number of patients screened, and the percentages of eligible patients recruited and retained at 6-month follow-up. In addition to the study documentation (AUDIT C screening questionnaires, expression of interest and consent forms), detailed logs documenting each patient approached and screened along with the outcome of screening, expression of interest and consent will be kept by each site and will be provided to the NCTU on a monthly basis. A number of tools will assess response variability in key measures proposed to be used in a future definitive trial where the primary outcome would be perioperative drinking status. Baseline alcohol consumption, health-related quality of life and joint functionality will be assessed by the full AUDIT [34], EQ-5D and Western Ontario and McMaster Universities Osteoarthritis Index (WOMAC) Functional Assessment score [46] respectively. Changes in these measures will be assessed with completion of the same measures at follow-up (1–3 days pre-surgery (AUDIT only), 6 weeks post-surgery and 6 months post intervention). Major and minor postoperative complications will be recorded by Clavien-Dindo classification [54] and the Postoperative Morbidity Survey (POMS) [55] collected at 3–5 days post-surgery.

Due to the low-risk nature of the study, adverse events will not be recorded. All serious adverse events (SAEs) excluding those which relate to pre-existing medical conditions, planned rehospitalisation and postsurgical complications which are covered by the trial outcome measures (specifically Clavien-Dindo and POMS) will be recorded by the PI or a delegate on a SAE form which will be sent to the senior trial manager, trial manager, chief investigator and the sponsor. For each SAE, full details in medical terms and a case description will be given along with the event duration, action taken, outcome and seriousness criteria causality in the opinion of the investigator and whether the event is considered expected or unexpected. All safety data collected will be reviewed by the Trial Steering Committee and Data Monitoring Committee.

### Follow-up

Baseline measures will be completed at the preoperative assessment visit. Patients will be followed up 1–3 days preoperatively either by telephone or in hospital if admitted the day before surgery; up to 5 days post-surgery while in hospital; at the 6 weeks post-surgery outpatient appointment or by telephone; and 6 months post intervention.

Study data will be entered into the trial MACRO™ database. Access to the MACRO™ database will be granted to site’s PIs, their delegated data entry personnel at sites and NCTU trial management team for monitoring purposes.

### Qualitative work

Qualitative, in-depth, one-to-one semi-structured interviews will be conducted with both patient and HCP participants. Patients who indicate at initial consent that they would be happy to participate in a qualitative interview will be asked again if they are willing to take part in an interview as part of the 6-month post-intervention follow-up. Patients still willing to participate in an interview will be provided with an interview-specific information sheet by email or post (dependent on patient preference) at least 24 h before the interview is due to take place. Following patient recruitment and intervention delivery, HCPs involved in screening and/or intervention delivery will also be invited to take part in a qualitative interview. These interviews will be conducted by a Research Associate (EL) and will use a semi-structured topic guide to explore HCP and patient experiences of being involved in the trial, the acceptability of screening and intervention tools/materials, barriers and facilitators to screening and intervention delivery and the feasibility of delivering screening and intervention in the preoperative assessment clinic. Topic guides will include key questions and prompts but will be left flexible enough for discussion to include issues of importance to the participant or issues arising from previous interviews. All interviews will be digitally recorded, with consent, and transcribed verbatim.

### Planned analysis

#### Statistical analysis

The measures collected at each time point are displayed in Table 3. Analysis will be descriptive in nature. The number of patients approached, screened, found to be eligible and recruited (as documented in screening logs) will be reported. Screening and eligibility rates will be assessed.

The primary feasibility outcome is recruitment and retention to 6-month follow-up. Systematic reviews and meta-analyses of alcohol brief intervention trials show that follow-up rates vary with mean follow-up rates of approximately 75% [56, 57] though rates of follow-up for older adult populations may be higher (e.g. [58]). It was therefore decided to allow for a loss to follow-up of 25%. Based on current recommendations for external pilot trials [59], 30 patients per arm at 6-month follow-up were required to estimate the critical parameters to the necessary degree of precision with a continuous primary outcome. The pilot trial will therefore aim to obtain data from 40 participants in each trial arm at baseline. As follow-up rates vary between trials of alcohol brief interventions, the target recruitment sample size will be kept under review, and recruitment will cease when we estimate that 60 patients will provide data at 6 months post intervention.

Secondary outcomes of compliance with randomisation (whether or not participants received the intervention when randomised to the intervention condition), and data completion of baseline measures will also be reported. Non-completers will be characterised.

The pilot feasibility trial will also assess performance of potential outcome measures for a definitive trial. We will ascertain data completeness of the study tools and any potential bias in the completion of follow-up data (assessing if completion rates on each clinical outcome are markedly different in either trial arm), to inform the choice of tools for a future trial.

The majority of data will be presented in simple descriptive tables presenting percentages, means and standard deviations or five-number summary (the minimum, first quartile, median, third quartile, and maximum) as appropriate, for each arm of the study. This information will be used to inform the design, choice of primary outcome, necessary sample size and approach to the analysis, of the future definitive trial.

### Qualitative analysis

Qualitative data from HCP and patient interviews will be used to assess the acceptability of screening, intervention and research methods. Anonymised transcripts will be analysed using Framework analysis [60]. This is a recommended approach for qualitative health research with objectives linked to quantitative investigation [61]. NVivo software will be used to aid indexing and charting. The data will be repeatedly read and coded independently by two researchers (EL, KH) within a framework of a priori issues and those identified by participants (patients or HCPs) or emerging from the data. Any divergence between coders will be discussed on an on-going basis to inform the analysis and resolve divergence in their interpretations of the data. Analysis will be discussed at regular meetings of the research team to identify areas for closer consideration (including negative case analysis) and to enhance credibility of the thematic framework and interpretation [61, 62].

Framework analysis of the qualitative data will explore influences on patient recruitment, implementation, receipt of the study interventions and data collection methods. Analysis of the likelihood of embedding study interventions in clinical practice (HCP data) will be informed by Normalisation Process Theory [63]. This model considers factors that affect implementation in four key areas; how people make sense of a new practice (coherence); the willingness of people to sign-up and commit to the new practice (cognitive participation); their ability to take on the work required of the practice (collective action); and activity undertaken to monitor and review the practice (reflexive monitoring). The approach is increasingly used in studies of the implementation of interventions in health care (www.normalizationprocess.org). Analysis will consider how well the behavioural intervention is introduced and incorporated for both patients and HCPs.

### Fidelity analyses

Consultations with a participating patient will be audio recorded to allow an assessment of skill acquisition and fidelity of delivery post-training. Audio recordings will be transcribed verbatim. A Research Associate (EL) trained in the use of the Behaviour Change Taxonomy Version 1 (BCT v1) and an expert coder (LA) will independently code all consultation transcripts to assess skill acquisition (for feedback purposes) and fidelity of delivery of each specific BCT. A coding frame/fidelity checklist based on the BCTv1 will be used to identify whether each BCT was delivered faithfully as planned. Where discrepancies in coding exist, the RA and expert coder will meet to resolve these via discussion. The percentage of positive agreement will be calculated to assess inter-rater reliability.

### Monitoring, audit and inspection

Monitoring will be performed by a combination of central review and site monitoring visits and external Data Monitoring Committee to ensure the study is conducted in accordance with GCP. Study site monitoring will be undertaken by Newcastle Clinical Trial Unit. The main areas of focus will include consent, serious adverse events, data completeness and accuracy and essential documents in study. The trial may be subject to audit by representatives of the Sponsor. Each investigator site will permit trial-related monitoring, audits and regulatory inspection including access to all essential and source data relating to the trial.

Data will be analysed by the Trial Statisticians and reported to an external independent DMC at least annually.

## Discussion

Existing research demonstrates the benefit of clinics dedicated to preoperative assessment [64] and a growing body of evidence points to the additional gains that can be made through implementation of prehabilitation targeting modifiable factors including physical activity, nutrition and smoking cessation [28, 65, 66]. Increased preoperative alcohol consumption is associated with an increased risk of postoperative complications [1, 2] thus interventions which reduce preoperative alcohol consumption may be one method of reducing surgical risk [3, 30]. While previous work has identified that alcohol screening and brief intervention is effective in other settings, especially primary care [36, 37], little is known about the feasibility of delivering these techniques in the preoperative setting. Significant uncertainties mean that proceeding immediately to a definitive trial would be a high risk.

Three key factors which restricted initial recruitment were identified and addressed during the feasibility work. Firstly, a number of patients demonstrated adverse responses to the term ‘risky drinking’ which previously appeared in the study title and documentation, this was amended so that wording focuses on the benefits of reducing alcohol consumption. Secondly, screening reactivity resulted in reductions in reported alcohol consumption at baseline, prior to confirmation in eligibility and meant that the number of patients who screened eligible, consented to the study, but did not go on to receive the intervention was higher than anticipated. For this reason eligibility for the RCT will be based on the initial screening score with later measures employed to track changes in alcohol consumption over the study period. Finally, allowing the patient too long to consider involvement in the study before obtaining ongoing expression of interest hindered the facilitation and organisation of intervention sessions, especially when patients were not contactable on the first attempt, and led to many patients forgetting about the study. Allowing patients a minimum of 24 h to consider their involvement means that the study information is fresh in their minds and facilitates effective coordination of preoperative assessment and intervention delivery. Further operational issues, specifically the availability of physical space and staff time in the clinics, were also identified but could not be fully addressed within the scope of this study as this would require a significant increase in financial and human resources. Further to this, obtaining research participation without an impact on patient care could be difficult if there is a temporary reduction in staff levels through sickness or absence, something which cannot be controlled. However, the pilot RCT implemented some strategies to reduce the impact of these issues. The burden on staff time and need for physical space to conduct study activities were reduced as much as possible by having research-specific activities (e.g. consent, randomisation 6-month follow-up) conducted by research rather than clinical staff and allowing screening and follow-up measures to be collected over the telephone rather than in person. In addition a ‘coping planning’ element was included in the revised HCP training and training manual. This provided specific examples of difficulties encountered during the feasibility trial and encouraged HCPs to pre-plan methods of coping should they encounter these difficulties (e.g. being short on time) as well as providing suggested coping methods if they could not come up with their own. The ongoing impact and potential mechanisms for overcoming these difficulties will be considered in the qualitative interviews with HCPs.

Quantitative findings will investigate the recruitment and retention of patient participants, comparing these to target rates as well as completion rates of planned outcome measures employed. This will allow conclusions about the feasibility of proceeding to a definitive RCT to be drawn and an accurate sample size calculation for a full RCT to be produced. Qualitative data and fidelity of delivery assessments from this pilot RCT will be used to further optimise intervention materials and methods, and to establish the acceptability of screening, intervention and research methods. In addition to informing a future definitive RCT this work may act as a model of risk reduction interventions targeting other health behaviours associated with post-operative complications such as smoking and physical activity.

### Current status of study

The trial commenced recruitment in July 2017.

## Abbreviations

AUDIT: Alcohol Use Disorders Identification Tool
BA: Brief Advice
BCT: Behaviour change technique
BI: Brief Intervention
CI: Confidence intervals
HCP: Healthcare professional
NCTU: Newcastle Clinical Trials Unit
OR: Odds ratio
PAC: Preoperative assessment clinic
POMS: Postoperative Morbidity Survey
RA: Research Associate
RCT: Randomised controlled trial
TAU: Treatment as usual
WOMAC: Western Ontario McMaster Universities Osteoarthritis Index

## References

[1] Oppedal K, Møller AM, Pedersen B, et al. Preoperative alcohol cessation prior to elective surgery. Cochrane Database Syst Rev. 2012;7. 10.1002/14651858.CD008343.pub2. 10.1002/14651858.CD008343.pub2 22786514

[2] Eliasen M, Grønkjær M, Skov-Ettrup LS (2013). Preoperative alcohol consumption and postoperative complications: a systematic review and meta-analysis. Ann Surg.

[3] Harris AHS, Reeder R, Ellerbe L (2011). Preoperative alcohol screening scores: association with complications in men undergoing total joint arthroplasty. J Bone Joint Surg Am.

[4] Rotevatn TA, Bøggild H, Olesen CR (2017). Alcohol consumption and the risk of postoperative mortality and morbidity after primary hip or knee arthroplasty—a register-based cohort study. PLoS One.

[5] Khuri SF, Henderson WG, DePalma RG (2005). Determinants of long-term survival after major surgery and the adverse effect of postoperative complications. Ann Surg.

[6] Abbott TEF, Fowler AJ, Dobbs TD (2017). Frequency of surgical treatment and related hospital procedures in the UK: a national ecological study using hospital episode statistics. Br J Anaesth.

[7] Lawrence VA, Hazuda HP, Cornell JE (2004). Functional independence after major abdominal surgery in the elderly. J Am Coll Surg.

[8] Moonesinghe SR, Harris S, Mythen MG (2014). Survival after postoperative morbidity: a longitudinal observational cohort study. Br J Anaesth.

[9] Belmont PJ, Goodman GP, Waterman BR (2014). Thirty-day postoperative complications and mortality following total knee arthroplasty: incidence and risk factors among a national sample of 15,321 patients. JBJS.

[10] Hunt LP, Ben-Shlomo Y, Clark EM (2013). 90-day mortality after 409 096 total hip replacements for osteoarthritis, from the National Joint Registry for England and Wales: a retrospective analysis. Lancet.

[11] Hunt LP, Ben-Shlomo Y, Clark EM (2014). 45-day mortality after 467 779 knee replacements for osteoarthritis from the National Joint Registry for England and Wales: an observational study. Lancet.

[12] Inneh IA, Lewis CG, Schutzer SF (2014). Focused risk analysis: regression model based on 5,314 total hip and knee arthroplasty patients from a single institution. J Arthroplast.

[13] (HSCIC) HaSCIC (2015). Hospital Episode statistics 2013/2014.

[14] Lloyd-Sherlock P, McKee M, Ebrahim S (2012). Population ageing and health. Lancet.

[15] Divo MJ, Martinez CH, Mannino DM. Ageing and the epidemiology of multimorbidity. Eur Respiratory Soc. 2014;44(4):1055–68. 10.1183/09031936.00059814 PMC4918092 25142482

[16] Pearse RM, Moreno RP, Bauer P (2012). Mortality after surgery in Europe: a 7 day cohort study. Lancet.

[17] Findlay G, Goodwin A, Protopapa K (2011). Knowing the risk; a review of the peri-operative care of surgical patients.

[18] Rose J, Weiser TG, Hider P (2015). Estimated need for surgery worldwide based on prevalence of diseases: implications for public health planning of surgical services. Lancet Glob Health.

[19] Cook JR, Warren M, Ganley KJ (2008). A comprehensive joint replacement program for total knee arthroplasty: a descriptive study. BMC Musculoskelet Disord.

[20] Goodnough LT, Maniatis A, Earnshaw P (2011). Detection, evaluation, and management of preoperative anaemia in the elective orthopaedic surgical patient: NATA guidelines. Br J Anaesth.

[21] Kothmann E, Batterham AM, Owen SJ (2009). Effect of short-term exercise training on aerobic fitness in patients with abdominal aortic aneurysms: a pilot study. Br J Anaesth.

[22] Tew GA, Moss J, Crank H (2012). Endurance exercise training in patients with small abdominal aortic aneurysm: a randomized controlled pilot study. Arch Phys Med Rehabil.

[23] Topp R, Swank AM, Quesada PM (2009). The effect of prehabilitation exercise on strength and functioning after total knee arthroplasty. PM&R.

[24] Santa Mina D, Clarke H, Ritvo P (2014). Effect of total-body prehabilitation on postoperative outcomes: a systematic review and meta-analysis. Physiotherapy.

[25] Carli F, Charlebois P, Baldini G (2009). An integrated multidisciplinary approach to implementation of a fast-track program for laparoscopic colorectal surgery. Can J Anesth.

[26] Carli F, Scheede-Bergdahl C (2015). Prehabilitation to enhance perioperative care. Anesthesiol Clin.

[27] Grocott MPW, Mythen MG (2015). Perioperative medicine: the value proposition for anesthesia?: a UK perspective on delivering value from anesthesiology. Anesthesiol Clin.

[28] Musallam KM, Rosendaal FR, Zaatari G (2013). Smoking and the risk of mortality and vascular and respiratory events in patients undergoing major surgery. JAMA Surg.

[29] Best MJ, Buller LT, Gosthe RG (2015). Alcohol misuse is an independent risk factor for poorer postoperative outcomes following primary total hip and total knee arthroplasty. J Arthroplast.

[30] Bradley KA, Rubinsky AD, Sun H (2011). Alcohol screening and risk of postoperative complications in male VA patients undergoing major non-cardiac surgery. J Gen Intern Med.

[31] Wilson GB, Wray C, McGovern R (2014). Intervention to reduce excessive alcohol consumption and improve comorbidity outcomes in hypertensive or depressed primary care patients: two parallel cluster randomized feasibility trials. Trials.

[32] Poon A, Owen J, Gijsbers AJ (1994). Identification of at-risk drinkers in an orthopaedic inpatient population. ANZ J Surg.

[33] Shourie S, Shourie S, Conigrave KM (2007). Pre-operative screening for excessive alcohol consumption among patients scheduled for elective surgery. Drug Alcohol Rev.

[34] Saunders JB, Aasland OG, Babor TF (1993). Development of the alcohol use disorders identification test (AUDIT): WHO collaborative project on early detection of persons with harmful alcohol consumption-II. Addiction.

[35] Kip MJ, Neumann T, Jugel C (2008). New strategies to detect alcohol use disorders in the preoperative assessment clinic of a German university hospital. J Am Soc Anesthesiol.

[36] Kaner EF, Beyer F, Dickinson HO, et al. Effectiveness of brief alcohol interventions in primary care populations. Cochrane Database Syst Rev. 2007;2. 10.1002/14651858.CD004148.pub3. 10.1002/14651858.CD004148.pub3 17443541

[37] Kaner E, Bland M, Cassidy P (2013). Effectiveness of screening and brief alcohol intervention in primary care (SIPS trial): pragmatic cluster randomised controlled trial. BMJ.

[38] Kaner E, Beyer, FR, Muirhead, C, Campbell, F, Pienaar, ED, Bertholet, N, Daeppen, J, Saunders, JB, Burnand, B. Effectiveness of brief alcohol interventions in primary care populations. Cochrane Database Syst Rev. 2018(Issue 2.):Art. No.: CD004148. 10.1002/14651858.CD004148.pub4 PMC6491186 29476653

[39] Beyer F, Lynch E, Kaner E (2018). Brief interventions in primary care: an evidence overview of practitioner and digital intervention programmes. Curr Addict Rep.

[40] Blow FC, Barry KL, Walton MA (2006). The efficacy of two brief intervention strategies among injured, at-risk drinkers in the emergency department: impact of tailored messaging and brief advice. J Stud Alcohol.

[41] Rosenberg J, Nielsen HJ, Rasmussen V (1999). Effect of preoperative abstinence on poor postoperative outcome in alcohol misusers: randomised controlled trial. BMJ.

[42] McQueen J, Howe TE, Allan L, et al. Brief interventions for heavy alcohol users admitted to general hospital wards. Cochrane Database Syst Rev. 2011;8(8). 10.1002/14651858.CD005191.pub3. 10.1002/14651858.CD005191.pub3 PMC10600352 21833953

[43] Shourie S, Conigrave KM, Proude EM (2006). The effectiveness of a tailored intervention for excessive alcohol consumption prior to elective surgery. Alcohol Alcohol.

[44] Brooks R, Group E (1996). EuroQol: the current state of play. Health Policy.

[45] Herdman M, Gudex C, Lloyd A (2011). Development and preliminary testing of the new five-level version of EQ-5D (EQ-5D-5L). Qual Life Res.

[46] Dowsey MM, Choong PFM. The utility of outcome measures in total knee replacement surgery. Int J Rheumatol. 2013;2013:506518. 10.1155/2013/506518 PMC3833283 24288541

[47] Coulton S, Perryman K, Bland M (2009). Screening and brief interventions for hazardous alcohol use in accident and emergency departments: a randomised controlled trial protocol. BMC Health Serv Res.

[48] Centre for Drug and Alcohol Studies (1993). The Drink-Less Programme.

[49] McAvoy B, Kaner E, Haighton K (1997). Drink-Less. Marketing a brief intervention package in UK general practice. Fam Pract.

[50] Longabaugh R, Woolard RE, Nirenberg TD (2001). Evaluating the effects of a brief motivational intervention for injured drinkers in the emergency department. J Stud Alcohol.

[51] Baird J, Longabaugh R, Lee CS (2007). Treatment completion in a brief motivational intervention in the emergency department: The effect of multiple interventions and therapists’ behavior. Alcohol Clin Exp Res.

[52] Fleming MF, Mundt MP, French MT (2002). Brief physician advice for problem drinkers: long-term efficacy and benefit-cost analysis. Alcohol Clin Exp Res.

[53] Kaner EFS, Dickinson HO, Beyer F (2009). The effectiveness of brief alcohol interventions in primary care settings: a systematic review. Drug Alcohol Rev.

[54] Dindo D, Demartines N, Clavien P-A (2004). Classification of surgical complications: a new proposal with evaluation in a cohort of 6336 patients and results of a survey. Ann Surg.

[55] Grocott MPW, Browne JP, Van der Meulen J (2007). The postoperative morbidity survey was validated and used to describe morbidity after major surgery. J Clin Epidemiol.

[56] Wilk AI, Jensen NM, Havighurst TC (1997). Meta-analysis of randomized control trials addressing brief interventions in heavy alcohol drinkers. J Gen Intern Med.

[57] Bertholet N, Daeppen J-B, Wietlisbach V (2005). Reduction of alcohol consumption by brief alcohol intervention in primary care: systematic review and meta-analysis. Arch Intern Med.

[58] Fleming MF, Manwell LB, Barry KL (1999). Brief physician advice for alcohol problems in older adults: a randomized community-based trial. J Fam Pract.

[59] Lancaster GA, Dodd S, Williamson PR (2004). Design and analysis of pilot studies: recommendations for good practice. J Eval Clin Pract.

[60] Srivastava A, Thomson SB (2009). Framework analysis: a qualitative methodology for applied policy research.

[61] Pope C, Ziebland S, Mays N (2000). Analysing qualitative data. Br Med J.

[62] Schlosser R (2002). On the importance of being earnest about treatment integrity. Augment Altern Commun.

[63] May CR, Finch T, Ballini L (2011). Evaluating complex interventions and health technologies using normalization process theory: development of a simplified approach and web-enabled toolkit. BMC Health Serv Res.

[64] Snowden CP, Anderson H (2012). Preoperative optimization: rationale and process: is it economic sense?. Curr Opin Anesthesiol.

[65] Fearon KC, Jenkins JT, Carli F (2013). Patient optimization for gastrointestinal cancer surgery. Br J Surg.

[66] Durrand JW, Batterham AM, Danjoux GR (2014). Pre-habilitation (i): aggregation of marginal gains. Anaesthesia.

